# Individual differences in inhibitory control may influence how endangered eels deal with river barriers during migration

**DOI:** 10.1007/s10071-026-02056-2

**Published:** 2026-03-07

**Authors:** Gaia De Russi, Mattia Lanzoni, Giuseppe Castaldelli, Cristiano Bertolucci, Angelo Bisazza, Tyrone Lucon-Xiccato

**Affiliations:** 1 Department of Life Sciences and Biotechnology, University of Ferrara, Ferrara, Italy; 2 Department of Environmental and Prevention Sciences, University of Ferrara, Ferrara, Italy; 3 Department of General Psychology, University of Padova, Padova, Italy

**Keywords:** *Anguilla anguilla*, Cognitive ecology, Cognitive variance, Conservation behaviour, Movement ecology

## Abstract

The European eel is currently facing a critical risk of extinction. One of the contributing factors is that its catadromous migration is often obstructed by artificial barriers such as dams. Migrating juvenile eels display a strong rheotactic behaviour. Therefore, we hypothesized that inhibitory control, the ability to suppress motivated behaviour, in this case rheotaxis, could facilitate the search for alternative routes when encountering a barrier. Using a detour test and a test in which a shelter was blocked by a transparent barrier, we demonstrated that migrating eels exhibit some degree of inhibitory control, with evidence of individual differences in performance. Crucially, individual differences in these inhibitory control tests significantly predicted how eels behaved in a simulated river system with barriers. Individual eels may adopt distinct strategies to overcome migration barriers and these differences might be partly driven by variations in inhibitory control.

**Supplementary Information:**

The online version contains supplementary material available at 10.1007/s10071-026-02056-2.

## Introduction

The European eel (*Anguilla anguilla*) is renowned for its extraordinary migration, spanning thousands of kilometres across the Atlantic Ocean to reach the European coasts, before continuing inland through freshwater rivers, sometimes for hundreds of kilometres (Cresci 2020; Van Ginneken and Maes 2005). During the freshwater stage of their migration, eels face the challenge of overcoming both natural and human-made barriers, which may prevent juveniles from reaching habitats suitable for their growth. It is estimated that in some regions, way more than 50% of rivers are obstructed by artificial structures such as dams (Belletti et al. 2020; Duarte et al. 2021), contributing to the European eel’s current serious risk of extinction (Jacoby et al. 2015; Liermann et al. 2012; Podda et al. 2022; Tamario et al. 2019).

During their upriver migration eels exhibit strong rheotaxis, a tendency to move against the current (Bolliet and Labonne 2008; Du Colombier et al. 2009). For instance, in a recent laboratory study, juvenile eels spent up to 50% of the time swimming against the direction of the water flow (De Russi et al. 2024). This strong rheotactic behaviour may aid in overcoming barriers, and several studies have examined this aspect (Du Colombier et al. 2009; Piper et al. 2012), often noting significant individual differences (De Russi et al. 2024; Mensinger et al. 2021; Podgorniak et al. 2016). However, an alternative migratory strategy could involve abandoning attempts to pass a barrier and rerouting to a different river or section of the river to find an alternative path. This would require inhibiting the strong rheotactic drive in the presence of a barrier.

In humans, the cognitive function involved in situations where a strongly motivated behaviour must be suppressed is commonly referred to as inhibitory control (Diamond 2013). Studies have shown that several species of teleost fish can solve typical inhibitory control tasks used in humans and other vertebrates (reviewed in Lucon-Xiccato 2024), although it remains unclear whether they do so using homologous or analogous cognitive functions. Another critical feature that characterises inhibitory control in fish is the marked inter-individual variation (Lucon-Xiccato et al. 2020a, b; Macario et al. 2021).

It is currently unknown whether eels exert inhibitory control during their migratory phase, and if so, to what extent. On the one hand, it is possible that they are so strongly motivated to migrate that they cannot inhibit their rheotactic drive. On the other hand, it could also be hypothesised that eels display inhibitory control, allowing for more flexible behaviour when encountering obstacles. In addition, eventual individual differences in eels’ inhibitory control may influence the strategies adopted by individual eels when encountering barriers during migration. To test these hypothesises, we assessed the eels’ inhibitory behaviour in a simulated migratory scenario, where their upstream movement was blocked and they were unable to continue forward. Moreover, we investigated whether they could solve tasks similar to those commonly used to assess inhibitory control in other animals. First, we used a detour test (Kabadayi et al. 2018), a paradigm in which a subject must detour around a barrier to reach a target behind it. We used water flow as a motivating stimulus and due to this, the barrier was made of grid rather than the transparent material typically used in such tests (Kabadayi et al. 2018). To complement this detour setup, we also used a shelter-seeking test in which the entrance to a refuge was blocked by a transparent barrier. Finally, by analysing individual performance across all three settings, we investigated how variation in inhibitory control influences the strategies eels use to approach and respond to barriers.

## Materials and methods

### Experimental design

Thirteen elver eels were collected during their upriver migration in the lower Po River (Italy). The sample was determined by specimen availability, which has substantially declined in the site in recent years (Aschonitis et al. 2017). The elvers were subsequently tested in the three behavioural assays (Online Resource 1). The order of the tests was kept constant across individuals to avoid different carryover effects that could influence individual differences’ measurements (Bell 2013). Elvers underwent the simulated upstream migration test first, as the subjects were collected during their migratory phase and we aimed to prioritize this ecologically relevant task. Subsequently, we conducted the detour test, which was based on an established procedure (Kabadayi et al. 2018; Lucon-Xiccato et al. 2017), and, finally, the shelter-seeking test, which we developed specifically for this species. A one-week interval was maintained between each test to minimize potential carryover effects. To enable consistent individual identification throughout the study, subjects were kept in separate housing tanks between tests.

### Simulated upstream migration

The test was performed in an apparatus presenting three corridors where a system of pumps generated a constant water flow through each of them (Figure S1a; Online Resource 1). Because we were interested in studying the behaviour of the eels when they encounter a barrier, each corridor was blocked by a net upstream that prevented the subjects from reaching the source of the flow. From video recordings, we scored the number of attempts made by each subject to swim through a corridor and the time spent in the corridor in each attempt and in the starting sector (BORIS software; Friard and Gamba 2016). For the analysis, we also calculated the percentage of attempts in which an individual chose a different corridor after exiting one (percentage of switches = number of switches to a different corridor / total number of attempts ⋅ 100). These three variables were expected to capture different aspects of eel migration behaviour, including motivation and persistence in attempting to pass a barrier and flexibility in exploring alternative routes.

### Inhibitory control test 1: detour

We used a modified version of the detour test, a paradigm widely employed across species (Kabadayi et al. 2018), including in teleost fish (Lucon-Xiccato et al. 2017; Gatto et al. 2018; Rochais et al. 2023; Santacà et al. 2019; Spiers and Gilbert 2015). Subjects were placed in the start sector of an apparatus where a system of pumps generated a constant water flow that motivated them to swim upstream. While swimming upstream, the eels encountered a C-shaped barrier made of grid netting (Figure S1b; Online Resource 1). Each subject underwent five trials on five consecutive days. From the video recordings of each trial, we scored whether the eel became blocked by the barrier, and if so, the duration of time it remained blocked. For the analyses, we considered three different variables. The first variable was the time spent in front of the barrier in the first trial, which, as it was based on the initial interaction with the paradigm, was expected to provide a measure of inhibitory control unaffected by prior experience. The second variable was the total time spent in front of the barrier across the five trials, providing a more comprehensive measure of eel performance over the entire experiment while incorporating potential effects of experience across trials. The final variable was the percentage of unsuccessful trials in which individuals failed to directly detour the barrier.

### Inhibitory control test 2: shelter-seeking

The second inhibitory control test involved a transparent barrier (Figure S1c). This new test was based on the tendency we observed in eels to seek shelter under objects (Online Resource 1). Following a training phase, we conducted an observation in which the openings of one shelter were blocked with transparent plastic. We scored the subjects’ ability to inhibit the tendency to enter the shelter measuring the time spent actively attempting to enter the blocked shelter and the number of entry attempts.

### Statistical analysis

Data analysis was performed using RStudio (ver. 2023.12.1). We used medians and 95% confidence intervals (CI) as descriptive statistics to deal with variables with non-normal distributions.

Initially, we focused on analysing subjects’ behaviour within each test. To assess whether subjects’ behaviour deviated from chance and to directly compare variables within-test, we conducted one-sample t-tests against chance and paired-sample t-tests. Temporal variations in behaviour were analysed through Linear Mixed-effects Models (LMMs) and a Generalized Linear Mixed-effect Model (GLMM), according to the variables’ distribution. In the LMMs, the response variable was the time of each trial or the number of attempts per minutes, the fixed effect was the attempt number or the trial, and subject ID was always fitted as random effect. In the GLMM, the number of attempts per minute was fitted as the response variable, while minute and subjects ID were fitted as fixed and random effect, respectively. Intra-class correlation coefficients (ICC) were also estimated to assess between-subject variance.

Subsequently, we performed a correlation analysis involving the variables from both the detour and shelter-seeking tests (detour test: time spent in the barrier in the first trial (s), total time spent in the barrier (s), unsuccessful trials (%); shelter-seeking tests: total time spent attempting to enter the shelter (s), shelter entry attempts (n)). This correlation analysis was performed to identify eventual individual differences in inhibitory performance. To handle outlier data points and focus on individual differences, we used the Spearman rank test (“cor.test” R function).

To reduce dimensionality and obtain a single measure of variability in performance on the inhibitory control tests, we then performed a Principal Component Analysis (PCA) based on Spearman’s rank correlation using the “PCA” function from the “EFA.dimension” library, focusing on the variables with significant correlations. As the total time spent in the barrier during the detour trials strongly covaried with the time spent in the barrier in the first trial, we excluded this variable from the PCA analysis to avoid data redundancy and inflation of the loadings. The “EFA_SCORE” function from the “EFA.dimension” library was then used to extract individual values associated with the new principal component (PC).

Finally, to test whether inhibitory control performance could predict individual eel behaviour in the simulated migration test, we used the PC obtained from the PCA in Generalised Linear Models (GLM) with Quasi-Poisson (“glm*”* library) and Beta (“betareg” library) error structure. We run three GLMs, with the following dependent variables: attempts to swim upstream (n), time spent in the corridors (%) and switches between corridors (%). When using the betareg function, the response variable was transformed by dividing it by 100. The variable “time spent in the corridors (%)” was computed as median to avoid effects of the distributions and as percentage for clear visualisation.

## Results

### Eels inhibited rheotactic behaviour

In the simulated migration test, eels exhibited the expected rheotactic response, entering one of the corridors directed upstream within 7.88 s (median; CI 5.96–11.84). Over the course of the test, eels spent significantly more time in the corridors than in the start sector (percentage of time spent in the corridor: 80.46%, CI 59.47–87.71; percentage of time spent in the start sector: 19.54%, CI 12.27–40.53; paired-sample t-test: t_12_ = 5.303, *p* < 0.001, Figure S2a, S2b), further suggesting a strong motivation to move upstream.

However, eels appeared capable of inhibiting their rheotactic response: when they could no longer continue upstream, all individuals rapidly left the end of the corridor and returned to the start sector. In most cases, this occurred in under 20 s (17.80 s, CI 15.16–19.96; 0.77%, CI 0.57–1.15, of the total time). Upon returning to the start sector, eels typically re-entered one of the corridors within a few seconds (6.08 s, CI 5.36–6.92). With each attempt, there was a progressive increase in the time spent in the start sector (LMM: *β* = 0.275, Χ^2^_1_ = 12.549, *p* < 0.001, Figure S2c) and the time spent in the corridors (LMM: *β* = 0.463, Χ^2^_1_ = 4.595, *p* = 0.032, Figure S2d): when eels entered one of these sectors, they were less likely to move to another sector at the end of the trial compared with the beginning of the trial.

There were significant individual differences in the time spent in the corridors during each attempt (ICC = 0.324), but not in the time spent in the start sector between attempts (ICC = 0.042). On average, an attempt to re-engage with a corridor occurred more than once per minute (number of attempts: 48, CI 25–57). In most cases, eels switched to a different corridor with each new attempt (percentage of switches: 80.00%, CI 71.70-86.89; one-sample t-test: t_12_ = 8.755, *p* < 0.001).

### Eels displayed inhibition in the inhibitory control tests

In the detour test, the eels were initially blocked in the barrier in the majority of trials (80%; CI 40, 100, Figure S3a). However, after remaining blocked in the barrier, eels rapidly detoured in all the trials (latency to detour: 4.05 s, CI 3.35–5.01, overall time spent in the barrier: 18.14 s, CI 6.06–21.67, Figure S3b), including the first trial (latency to detour in the first trial: 3.18 s, CI 1.81–31.15, Figure S3c). There was no evidence of improvement in the latency to detour across trials (*β* = 0.150, Χ^2^_1_ = 0.138, *p* = 0.711, Figure S3d) nor of significant individual differences (ICC = 0.025).

In the shelter-seeking test, subjects made approximately 20 (CI 16–27; Figure S3e) shelter entry attempts and spent overall less than a minute doing so (49.98 s, CI 37.16–87.76; Figure S3f). The length of the attempts did not significantly vary over time (LMM: *β* = 0.031, Χ^2^_1_ = 2.233, *p* = 0.136; Figure S3g), but their number significantly decreased during the test (GLMM: *β* = -0.079, Χ^2^_1_ = 18.490, *p* < 0.001, Figure S3h). We did not find strong individual differences in the time spent attempting to enter the shelter (ICC = 0.246), nor in the number of shelter entry attempts (ICC = 0.016).

### Eels displayed individual differences in inhibitory control tests

**Fig. 1:**
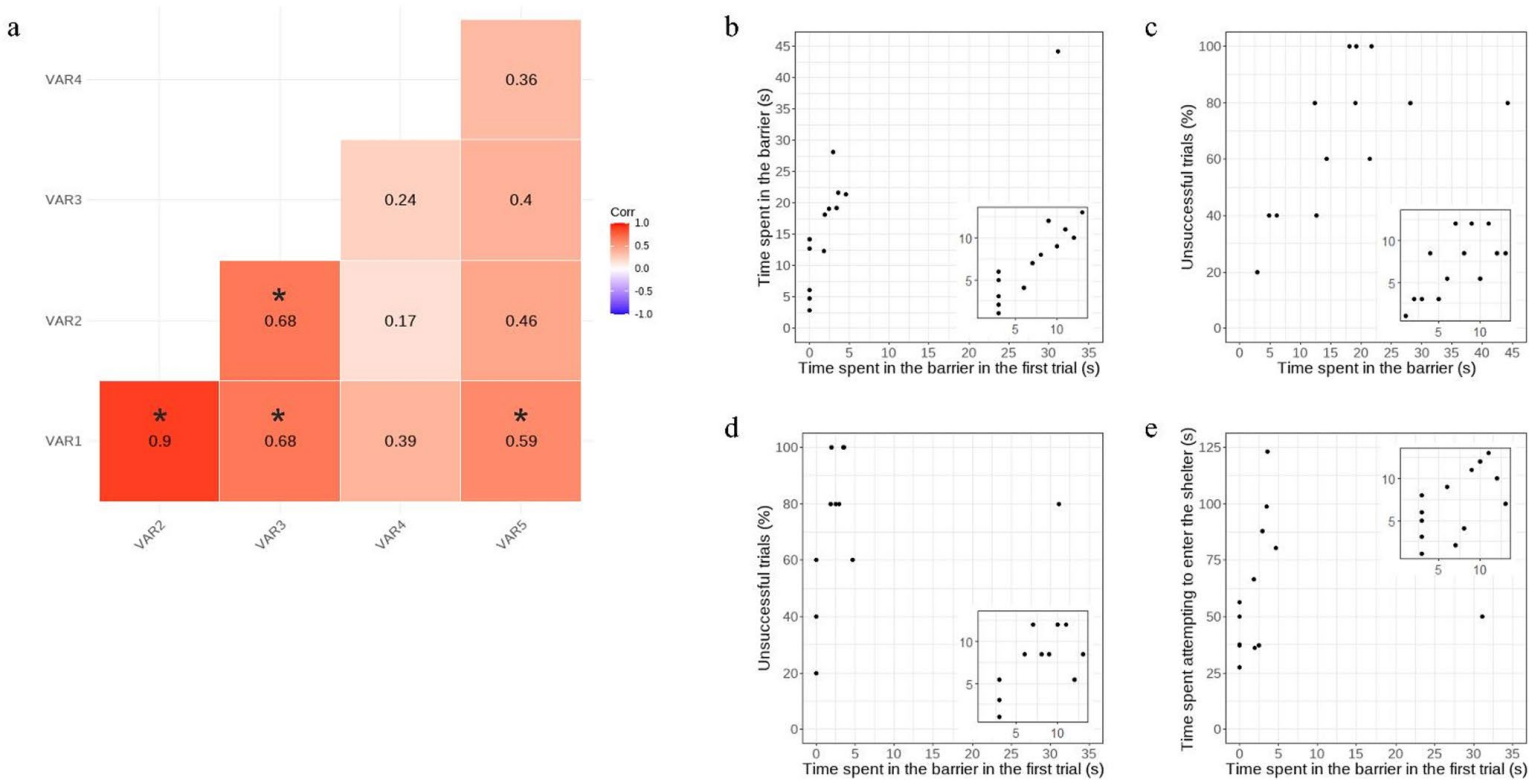
Covariation among inhibitory control measures. **a**) Correlation matrix among the inhibitory control variables collected in the detour and the shelter seeking test; VAR1: time spent in the barrier in the first trial (s), VAR2: time spent in the barrier (s), VAR3: unsuccessful detour trials (%), VAR4: shelter entry attempts (n), VAR5: time spent attempting to enter the shelter (s); asterisks indicate significant correlations. **b**-**e**) Scatterplots of the significant correlations between inhibitory control variables (note that the analyses used non-parametric tests to handle the outliers). The internal graphs represent the respective correlations with the data transformed into ranks

We found significant covariations among the variables collected in the detour and the shelter-seeking test (Fig. 1a). Within the detour test, the time spent in the barrier, both total and in the first trial, and the unsuccessful trials were positively correlated (time spent in the barrier – time spent in the barrier in the first trial: ρ = 0.899, *p* < 0.001; Fig. 1b; time spent in the barrier – unsuccessful trials: ρ = 0.680, *p* = 0.011; Fig. 1c; time spent in the barrier in the first trial – unsuccessful trials: ρ = 0.682, *p* = 0.010; Fig. 1d), suggesting that individuals differ in inhibitory performance.

Interestingly, we also detected a significant covariation between variables from the two tests: the time spent in the barrier in the first trial of the detour test was positively correlated with the time spent attempting to enter the shelter (ρ = 0.593, *p* = 0.033; Fig. 1e). This suggested that a single dimension of individual variation underlined part of the performance in the two inhibitory control tests. The PCA analysis to quantify this dimension detected a significant first component (PC1, eigenvalue = 2.364) accounting for 59.11% of total variance, negatively loaded by the four variables (time spent in the barrier in the first trial: -0.895; unsuccessful trials: -0.779; time spent attempting to enter the shelter: -0.777; shelter entering attempts: -0.595).

### Individual differences in inhibitory control predicted behaviour during simulated migration

**Fig. 2:**
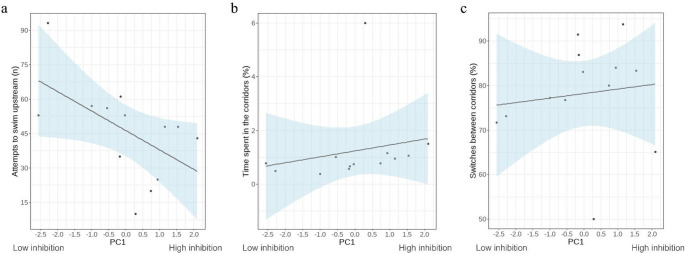
Relationship between inhibitory control and migratory behaviour. Scatterplot of the principal component (PC1) describing individual variation in inhibitory control versus (**a**) attempts to swim upstream (n), (**b**) time spent in the corridors (%), and (**c**) switches between corridors (%). PC1 is negatively loaded by time spent in the barrier in the first detour test trial (s), unsuccessful detour trials (%), time spent attempting to enter the shelter (s), and shelter entry attempts (n). The lines and shaded areas are predicted by GLM

The main dimension of individual differences in inhibitory control identified in the previous tests (PC1) significantly predicted the number of attempts to swim upstream in the migration experiment (*β* = -0.175, *p* = 0.047; Fig. 2a) but not the time spent in the corridors in each attempt (*β* = 0.164, *p* = 0.232, Fig. 2b) and the percentage of switches between corridors (*β* = 0.094, *p* = 0.448, Fig. 2c).

## Discussion

Migrating eel elvers are known to display strong rheotactic behaviour. Our study shows that this behaviour can be inhibited when eels encounter barriers that prevent upstream movement. Interestingly, our results also highlight individual variation in this aspect of migratory behaviour, which appears to be predicted by differences in performance on inhibitory control tasks.

In the simulated migration setting in the laboratory, elvers inhibited their rheotactic behaviour, rapidly interrupting unsuccessful attempts to swim upstream. This suggests that, in nature, when faced with a barrier preventing migration, the eels possess the ability to swim back and search for an alternative passage. This is remarkable given the strong rheotaxis observed in eels (De Russi et al. 2024; Edeline et al. 2005; Linton et al. 2007), which might have suggested that at least their migratory behaviour would be difficult to inhibit. Eels also displayed inhibition in the other two tests developed based on standard laboratory procedures. In the detour test, the eels were capable of avoiding the barrier with a performance comparable to what found for other teleost species (Lucon-Xiccato et al. 2017). In the second test, which used a transparent barrier in the context of entering a shelter, the eels were also able to inhibit their behaviour as seen by the decrease of attempts over time. These data suggest that eels possess some form of inhibitory control, as previously reports for a range of teleost fish (reviewed in Lucon-Xiccato 2024) as well as birds and mammals (MacLean et al. 2014).

Interestingly, we found substantial evidence of individual variation. In the simulated migration experiment, different individuals obtained significantly different scores, and in the detour test, different scores were correlated. Moreover, eels that were more persistent in trying to pass through the barrier and generally performed worst in the detour test, similarly showed greater persistence in attempting to enter the shelter blocked by the transparent wall. This aligns with observations in other teleost fish, which show substantial interindividual variation and intertask correlations in inhibitory control performance (Lucon-Xiccato et al. 2020a, b; Macario et al. 2021; Montalbano et al. 2020). Notably, in our study, individual variation in eels emerged from different contexts, rheotaxis and attempts to reach a shelter, in which different motivational factors were expected to be involved. Therefore, an explanation based on individual variation in motivation seems unlikely for our findings because it would involve two different motivational factors that are coincidentally associated in their interindividual variation. A more parsimonious interpretation would be that a single underlying cognitive function affects inhibition in both tasks. This interpretation is further supported by the presence of a principal component explaining approximately 60% of the variation in the two inhibitory control tests, further suggesting that eels display individual differences in their capacity to exert inhibitory control.

Critically, the principal component describing variation in the two inhibitory control tests significantly predicted the behaviour of eels in the simulated upriver migration setting. Specifically, individuals that had more difficulty detouring around the barrier and blocking attempts to enter the shelter also attempted more times to swim upriver. While it is difficult to generalise laboratory studies to natural situations, and in our case further caution is warranted by the sample size, our findings suggest that individual differences in inhibitory control may influence eels’ behaviour when encountering barriers, shaping strategies and success in overcoming naturally occurring obstacles such as waterfalls. This would represent one of the first documented links between inhibitory control and an aspect directly related to animal fitness (Coomes et al. 2022; Fichtel et al. 2023; Rochais et al. 2023). Moreover, it is worth considering that our findings could also apply to eels tackling human-made barriers, such as dams. Variation in inhibitory control may play a role in how successfully eels cope with these anthropogenic factors threatening the species. More generally, cognition may represent a neglected axis of biological diversity that is relevant to eels and other migratory species.

It would be interesting to understand the causes of this individual variation in eels’ inhibitory control. Eels are known to display substantial phenotypic plasticity (Capoccioni et al. 2014; De Meyer et al. 2016). Considering that inhibitory control is plastic in other fish species and varies based on individual experiences (Lucon-Xiccato et al. 2022), we speculate that phenotypic plasticity might in part determine the individual differences observed in eels. However, evidence suggests that genetic factors also contribute to individual differences in inhibitory control in fish (Lucon-Xiccato and Bertolucci 2020), suggesting that the variation observed in our study is, at least in part, the result of selective processes. While some of these processes may be unrelated to migration (Madden et al. 2018; Vinogradov et al. 2025), we cannot exclude direct selection for variability in inhibitory control specifically associated with migration. Indeed, such variation among individual eels’ migration behaviour may be beneficial. For example, individuals with different inhibitory control may display different persistence in attempting to pass a barrier and different tendency to reroute, which may help distribute individuals across a broader range of environments, reducing intraspecific competition and increasing the likelihood of finding suitable habitats for settlement. Preliminary sampling in our system appears to support this possibility of adaptive spatial sorting, as we collected only eels with specific cognitive and behavioural phenotypes in the initial section of the river basin (De Russi et al. 2024). However, more extensive sampling of natural populations is required to confirm this hypothesis.

## Supplementary Information

Below is the link to the electronic supplementary material.

Supplementary Material 1

Supplementary Material 2

## Data Availability

Data and code are submitted as supplementary material (Online Resource 2).

## Declarations

### AI use

We have not used AI-assisted technologies in creating this article.
