# Supplementary material for: Individual differences in inhibitory control may influence how endangered eels deal with river barriers during migration
Source: Anim Cogn. 2026 Mar 7;29(1):32. doi: 10.1007/s10071-026-02056-2 (PMC12979361; doi:10.1007/s10071-026-02056-2)

Individual differences in inhibitory control may influence how endangered eels deal with river barriers during migration

Gaia De Russi[1,*], Mattia Lanzoni[2], Giuseppe Castaldelli[2], Cristiano Bertolucci[1], Angelo Bisazza[3], Tyrone Lucon-Xiccato[1]

[1]Department of Life Sciences and Biotechnology, University of Ferrara, Ferrara, Italy.

[2]Department of Environmental and Prevention Sciences, University of Ferrara, Ferrara, Italy.

[3]Department of General Psychology, University of Padova, Padova, Italy.

*Correspondence: gaia.derussi@unife.it

This PDF file includes:

Extended Materials and Methods

Supplementary Figure S1-S3

Extended Materials and Methods

Subjects' husbandry

After collection, the eels were transferred to our university laboratory in aerated tanks and upon arrival were slowly habituated to salt water from the facility (Sea Salt, Aquaforest, Poland) adjusted to match the salinity of the sampling site (30.0 ppm). The following day, the subjects were individually moved into opaque plastic tanks (30 × 40 × 22 cm) where they were kept for the first part of the study. Water was kept at a temperature of 15 ± 1 °C. A white LED strip (TMR, distributed by ELCART, Italy; 0.031 W/m^2) lit each tank from above with a photoperiod of 12 h: 12 h light: dark. Each tank contained an aerator and a native plant to be used as shelter. The tanks were cleaned daily from food and faecal residuals, and the water was partially changed every other day. Subjects were fed alternatively with *Artemia salina* (20-30 nauplii) and defrosted chironomids larvae (*at libitum*), while once a week they were offered live zebrafish fry (age under 5 days post fertilisation). Testing was performed in the morning one hour after the lights had been switched on, while food was administered at the end of the daily testing session.

Simulated upstream migration

The apparatus (1000 × 30 × 45 cm; Figure S1A) was made of green plastic and filled with 10 cm of water. On one side, the apparatus featured three corridors (40 × 6 cm), which were accessible from an initial start sector (20 × 30 cm). The apparatus was lit from above with two fluorescent tubes (15 W, natural light, Sylvania GRO-LUX, Milano, Italy). The subject was netted from its housing tank and moved to the apparatus using a water-filled container. The container was then gently emptied into the start sector, from which the subject could freely attempt to swim upstream through the corridors. However, each corridor ended with a grid net that allowed water to pass but could not be physically overcome by the eels. The behaviour of

each subject was recorded from above using a video camera (Cx405, Sony Europe B.V., United Kingdom) for 40 minutes. After testing, subjects were returned to their housing tanks.

Inhibitory control test 1: detour

The apparatus (1000 × 30 × 45 cm; Figure S1B) was made of green plastic and filled with 10 cm of water. As in the previous apparatus, water pumps ensured constant water flow, which served to trigger the eels' rheotactic response and encouraged them to leave the start sector. A C-shaped barrier (14 × 4 cm), made of grid netting, was positioned 4 cm from the start sector. The apparatus was lit from above with two fluorescent tubes (15 W, natural light, Sylvania GRO-LUX, Milano, Italy). For testing, the subject was netted from its housing tank, moved to the apparatus in a water-filled container, and gently placed into the start sector, from which it could freely attempt to swim upstream. A trial was considered complete when the subject successfully detoured around the barrier and reached the source of the water flow. The behaviour of each subject was recorded from above using a video camera (Cx405, Sony Europe B.V., United Kingdom). After testing, the subjects were returned to their housing tanks.

Inhibitory control test 2: shelter-seeking

One week before the start of the training phase, the subjects were individually moved into larger housing tanks (60 × 40 × 40 cm; Figure S1C), where the experiment was also conducted. Two trapezoidal solid plastic barriers divided the tank into two equal sections, connected by a 13 × 5 cm corridor. Each section contained an identical plastic shelter, created by cutting a 10 cm pipe (⌀ = 2.5 cm) in half. The shelters were placed at 2 cm from the shorter tank wall, centred, with their openings aligned with the corridor. The subjects were trained twice per day for three days to switch from one shelter to the other by removing the one they were hidden in. Although we did not establish a formal criterion for passing the training phase,

all subjects were able to locate and use the second shelter in each training trial. Therefore, all individuals were equally familiar with the task at the end of the training phase. After the sixth trial, the previously removed second shelter was not placed back in the tank, to force the subjects to hide in the remaining one. The following day, we proceed with the test phase to measure inhibitory control, introducing as the second shelter one that had both its entrances blocked by transparent plastic. We removed the shelter in which the subject was hidden and recorded (Cx405, Sony Europe B.V., United Kingdom) its behaviour for the following 12 minutes.

Supplementary figures

Figure S1. Aerial view of the apparatuses used to study eels' behaviour. a) Simulated upstream migration test, b) detour test, and c) shelter seeking test.

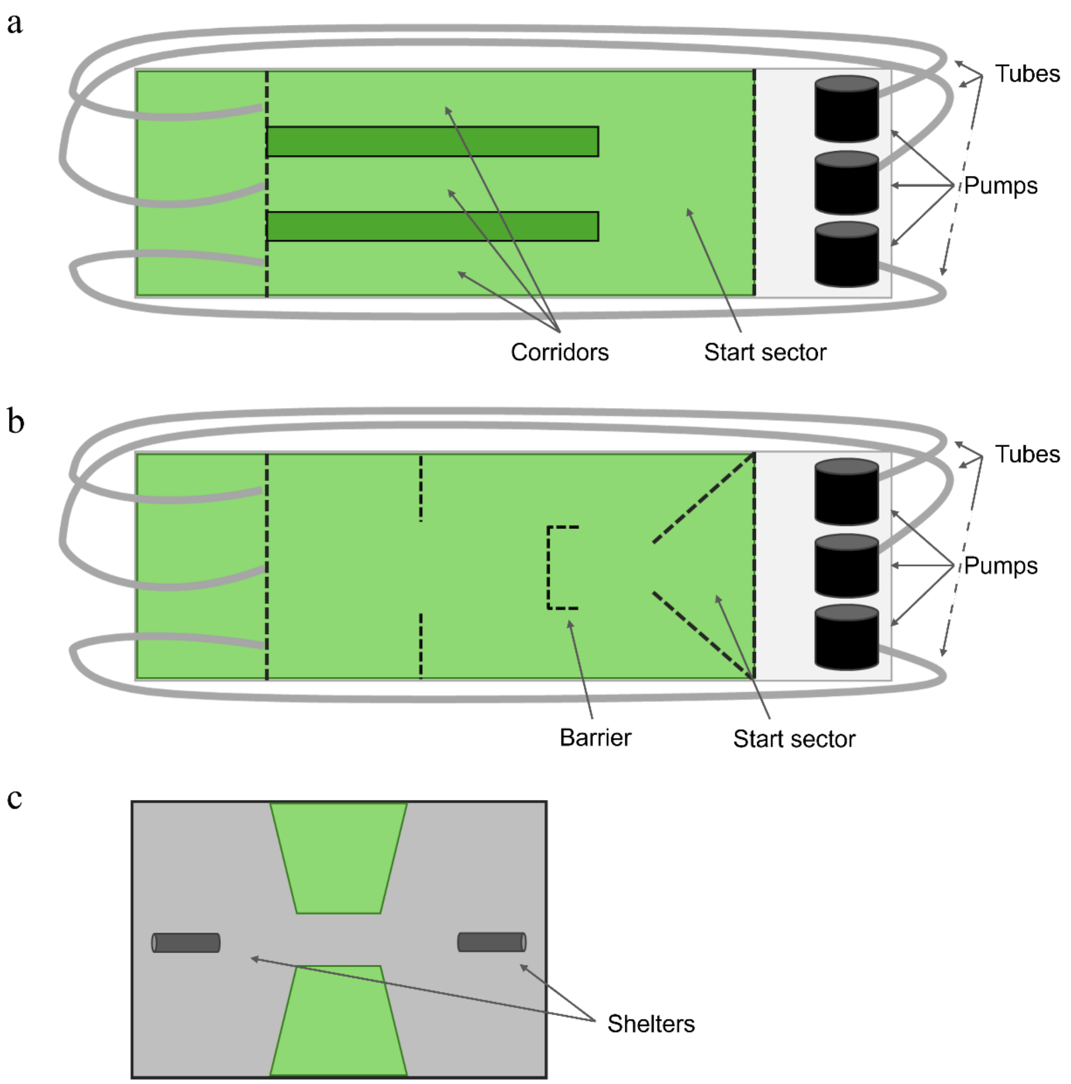

Figure S2. Eels in the rheotactic behaviour in the simulated migration test. Frequency distribution plots of time spent by the subjects a) in the corridors (%) and b) in the starting sector (%); bars represent observed data frequency and curves represent probability density functions. c) Time spent in the corridors and d) time spent in the starting sector versus attempt number; points and shaded areas represent respectively means and 95% confidence intervals predicted by the LMMs.

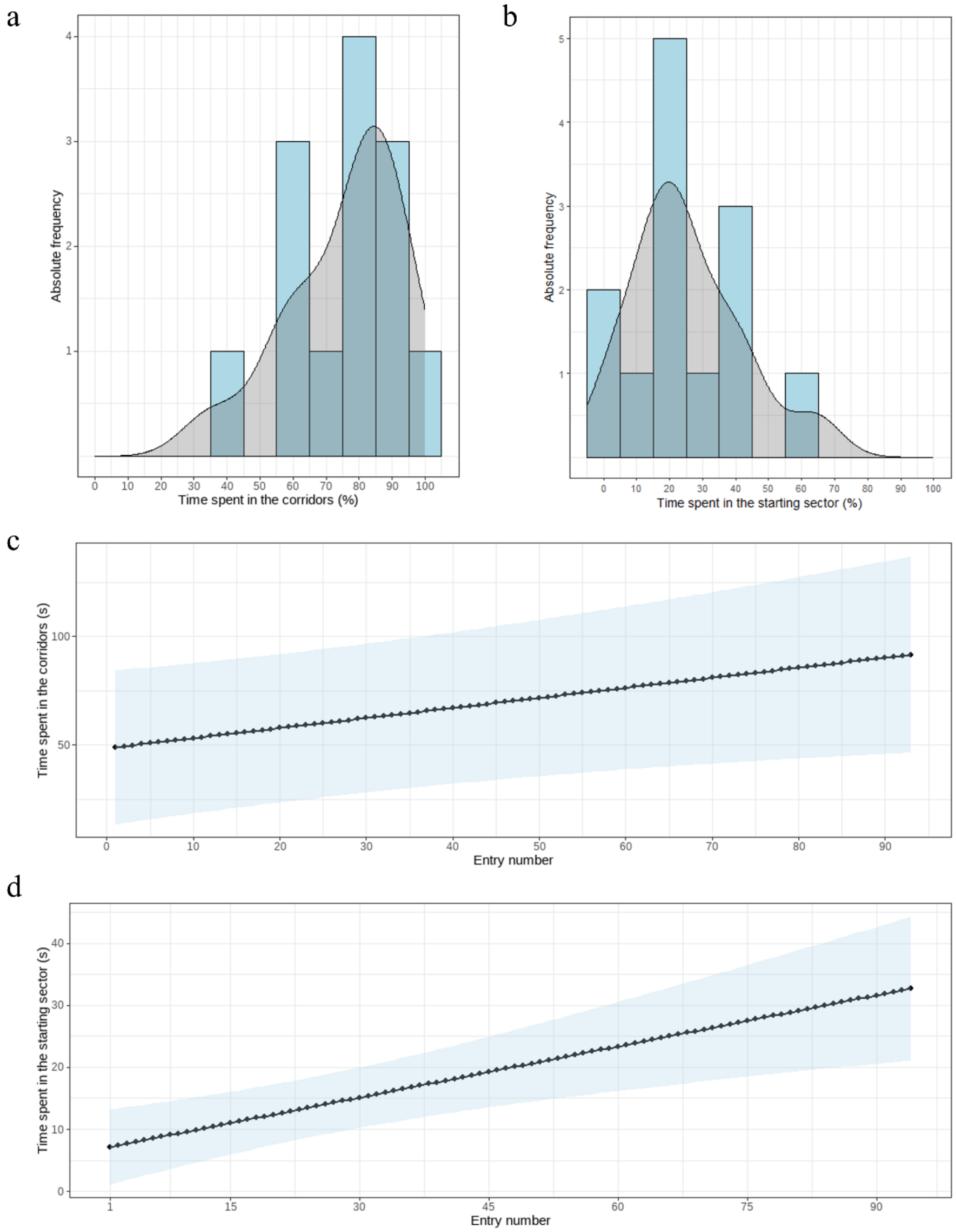

Figure S3. Performance of eels in the inhibitory control tests. Frequency distribution plots of a) unsuccessful detour trials (%), b) overall time spent in the barrier (s), c) time spent in the barrier in the first trial (s), e) shelter entry attempts (n), and f) time spent attempting to enter the shelter (s); bars represent observed data frequency and curves represent probability density functions. Temporal trend of d) time spent in the barrier in consecutive detour trials (s), g) shelter entry attempts length (s) and h) shelter entry attempts (n); points and shaded areas represent respectively means and 95% confidence intervals predicted by LMMs and GLMM.

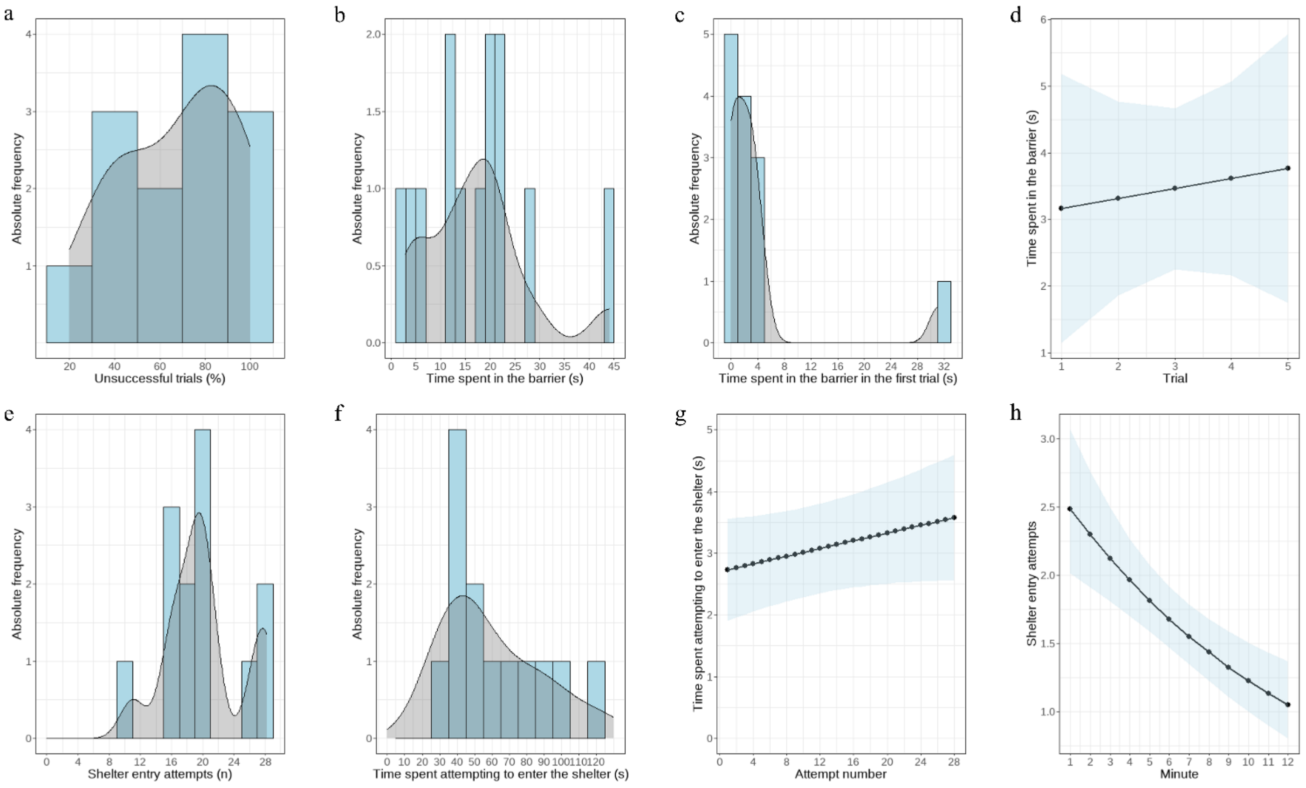

Supplement: Supplementary file 2 — Supplementary Material 2 [file 10071_2026_2056_MOESM2_ESM.pdf]
